# Plant Immune Responses to Parasitic Nematodes

**DOI:** 10.3389/fpls.2019.01165

**Published:** 2019-09-26

**Authors:** Kazuki Sato, Yasuhiro Kadota, Ken Shirasu

**Affiliations:** ^1^RIKEN Center for Sustainable Resource Science, Yokohama, Japan; ^2^Graduate School of Science, University of Tokyo, Bunkyo, Japan

**Keywords:** pattern-triggered immunity, NLR-triggered immunity, anti-nematode enzymes, anti-nematode compounds, cell wall reinforcement, reactive oxygen species, nitric oxide, hypersensitive response cell death

## Abstract

Plant-parasitic nematodes (PPNs), such as root-knot nematodes (RKNs) and cyst nematodes (CNs), are among the most devastating pests in agriculture. RKNs and CNs induce redifferentiation of root cells into feeding cells, which provide water and nutrients to these nematodes. Plants trigger immune responses to PPN infection by recognizing PPN invasion through several different but complementary systems. Plants recognize pathogen-associated molecular patterns (PAMPs) sderived from PPNs by cell surface–localized pattern recognition receptors (PRRs), leading to pattern-triggered immunity (PTI). Plants can also recognize tissue and cellular damage caused by invasion or migration of PPNs through PRR-based recognition of damage-associated molecular patterns (DAMPs). Resistant plants have the added ability to recognize PPN effectors *via* intracellular nucleotide-binding domain leucine-rich repeat (NLR)-type immune receptors, leading to NLR-triggered immunity. Some PRRs may also recognize apoplastic PPN effectors and induce PTI. Plant immune responses against PPNs include the secretion of anti-nematode enzymes, the production of anti-nematode compounds, cell wall reinforcement, production of reactive oxygen species and nitric oxide, and hypersensitive response–mediated cell death. In this review, we summarize the recognition mechanisms for PPN infection and what is known about PPN-induced immune responses in plants.

## Introduction

Plant-parasitic nematodes (PPNs) are among the most devastating agricultural pests worldwide with an annual global crop loss estimated at about 80 billion USD (Jones et al., 2013). PPNs infect a broad host range of commercially important crop families such as the Solanaceae (tomato, potato, pepper), Fabaceae (soybean), Malvaceae (cotton), Amaranthaceae (sugar beet), and Poaceae (syn. Gramineae; rice, wheat, maize). In general, the economically important PPNs have a broad host range and are highly virulent. PPNs may possess sophisticated virulent strategy as they can infect many plants without inducing strong immune responses (Warmerdam et al., 2018). This characteristic feature makes it difficult to isolate mutants of *Arabidopsis thaliana* that are defective in immunity against PPNs. However, recent progress in plant and nematode genomics has opened a way to understanding the plant’s mechanisms for recognizing PPN infection. There is now a large body of work surrounding the immune, tolerance, and susceptible responses of plant species to nematode infection (summarized in **Supplementary Table 1**). In this review, we summarize the known plant recognition mechanisms for PPN infection, and the host immune responses to PPN. In addition, we discuss how different recognition systems activate different immune responses.

## PPN Life Cycles

**Figure 1:**
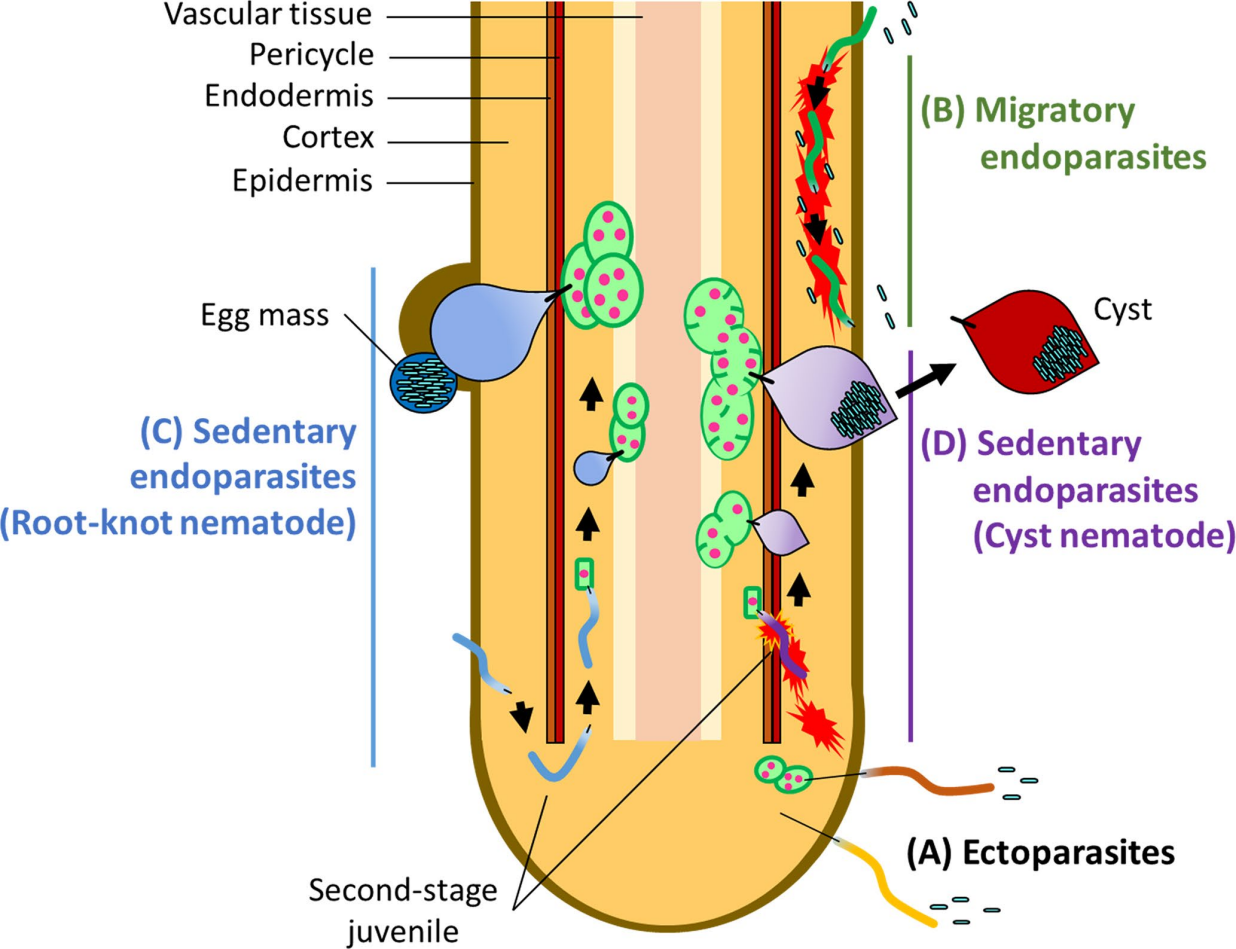
Infection strategies of PPNs **(A)** Ectoparasites take up nutrients from plant cells without invading the plant root. Some ectoparasites such as needle nematodes (*Longidorus* spp.) and dagger nematodes (*Xiphinema* spp.) induce the formation of nurse cells which extends the period of feeding. **(B)** Migratory endoparasites move through inside of the root tissues causing destruction *en route* and feed on plant tissues. Sedentary endoparasites include the root-knot nematodes (RKNs), *Meloidogyne* spp. and the cyst nematodes (CNs), including *Globodera* spp. and *Heterodera* spp. **(C)** Second-stage RKN juveniles enter the root near the root-tip then migrate intercellularly to the vascular cylinder where they reprogram root tissues into giant cells. After establishment of giant cells, RKN juveniles become sedentary and take up nutrients and water through a feeding stylet. Adult RKN females form an egg mass on or below the root surface. **(D)** Second-stage juveniles of the CNs move inside of the root intracellularly, causing destruction of plant tissues as they go, and establish syncytia in the vascular tissues as feeding cells. CN juveniles also become sedentary and start feeding from syncytia. Adult CN females retain eggs inside of the body, which forms a cyst after death.

PPNs are divided into three major groups according to feeding behavior: ectoparasitic, semi-endoparasitic, and endoparasitic (Decraemer and Hunt, 2013; Palomares-Rius et al., 2017; Smant et al., 2018). Ectoparasitic nematodes spend their entire life cycle outside of the host, with the only physical contact being the insertion of a long and rigid feeding stylet (**Figure 1A**). Semi-endoparasitic nematodes penetrate roots to feed, with its posterior part remaining in the soil. Endoparasitic nematodes completely enter the root and feed on internal tissues. Each of these feeding types is further divided into either migratory or sedentary lifestyles. For example, migratory endoparasites (e.g., the root-lesion nematodes *Pratylenchus* spp., and the burrowing nematodes *Radopholus* spp.) migrate through root tissues to feed on plant cells, causing damage to tissues as they migrate (**Figure 1B**), whereas sedentary endoparasites move into the vascular cylinder and induce redifferentiation of host cells into multinucleate and hypertrophic feeding cells. The two main PPNs in the sedentary group are the root-knot nematodes (RKNs) in the genus *Meloidogyne*, and the cyst nematodes (CNs) including the genera *Globodera* and *Heterodera* (**Figures 1C, D**). RKNs and CNs are the most devastating nematodes in the world (Jones et al., 2013).

Both RKNs and CNs induce host-cell redifferentiation to establish feeding cells for own development and reproduction, but in two different ways. Infective RKN juveniles enter near the root-tip and migrate intercellularly to the vascular cylinder where feeding cells are formed. Once RKNs enter a favorable location, they induce the redifferentiation of plant cells into multinucleate giant cells by repeated nuclear divisions without cytoplasmic division (Abad et al., 2009; Escobar et al., 2015). About 4–6 weeks after infection, the pear-shaped mature adult RKN female lays eggs in a gelatinous egg mass on or below the surface of the root (Abad et al., 2009; Escobar et al., 2015). RKNs exhibit variable reproduction modes such as amphimixis, facultative parthenogenesis and obligate parthenogenesis. In particular, the most devastating RKN species, *Meloidogyne incognita*, *Meloidogyne arenaria*, and *Meloidogyne javanica*, reproduce by obligate parthenogenesis and males appear to have no role in reproduction (Castagnone-Sereno, 2006). CN juveniles enter the root and move intracellularly into the vascular cylinder where, unlike RKNs, they induce syncytia through the local dissolution of cell walls and protoplast fusion of neighboring plant cells. Hundreds of eggs are produced inside of the female body after mating. When the female dies, its body forms a cyst, which can protect the eggs for many years in the soil (Bohlmann and Sobczak, 2014; Bohlmann, 2015). Both RKNs and CNs secrete virulence effectors through a stylet to manipulate host cells for establishing feeding cells. PPNs secrete effectors include cell wall degrading enzymes, inhibitors of anti-nematodal plant enzymes, plant immune signaling suppressors, and proteins required for the establishment of feeding cells (Davis et al., 2008; Gheysen and Mitchum, 2011; Hewezi and Baum, 2013; Goverse and Smant, 2014; Smant et al., 2018; Mejias et al., 2019).

## Recognition of PPNs

In general, pathogens are perceived by several different recognition systems in plants (Jones and Dangl, 2006; Dodds and Rathjen, 2010). The first recognition system is mediated by the perception of pathogen-associated molecular patterns (PAMPs) (e.g., bacterial flagellin, fungal chitin) and damage-associated molecular patterns (DAMPs) released by the disrupted host plant tissues. PAMPs and DAMPs are perceived by cell surface–localized pattern recognition receptors (PRRs), leading to pattern-triggered immunity (PTI) (Boutrot and Zipfel, 2017; Hou et al., 2019). Plant PRRs are usually either receptor-like kinases (RLKs) or receptor-like proteins (Boutrot and Zipfel, 2017). Successful pathogens secrete effector proteins into host apoplast and cytoplasm to interfere with recognition and immune signaling. In resistant plants, however, these effectors are often recognized by intracellular nucleotide-binding domain leucine-rich repeat (NLR)-type immune sensors, leading to NLR-triggered immunity (Cui et al., 2015). The N-terminus of NLR proteins usually contains a toll-interleukin 1 receptor (TIR) domain or coiled coil (CC), which are used to classify NLR proteins into two subgroups TIR-NLRs and CC-NLRs. In addition, some PRRs in resistant plants also recognize apoplastic effectors to induce PTI.

PPNs are known to induce PTI in plants. For example, ascaroside, an evolutionarily conserved nematode pheromone, is the first and only nematode PAMP identified so far (Manosalva et al., 2015). Ascr#18, the most abundant ascaroside in PPNs, activates typical plant immune responses, such as mitogen-activated protein kinases, PTI-marker gene expression, and salicylic acid– and jasmonic acid (JA)–mediated defense signaling pathways. Importantly, treatment with Ascr#18 increases resistance to both RKNs and CNs in *Arabidopsis*. Moreover, Ascr#18 is also recognized by tomato, potato, and barley, suggesting that the recognition of Ascr#18 is well conserved in both monocots and dicots. However, the corresponding PRR for recognizing Ascr#18 has not yet been identified. The first identified PRR involved in the induction of PTI in response to a PPN-derived molecule is a leucine-rich repeat (LRR)-RLK encoded by *Arabidopsis Nilr1* (*nematode-induced LRR-RLK 1*) (Mendy et al., 2017). NILR1 was isolated as an essential component for recognizing “NemaWater,” an aqueous solution incubated with infective-stage juveniles of CN (*Heterodera schachtii*) and RKN (*M. incognita*) as PTI inducers. Interestingly, the extracellular receptor domain of NILR1 is widely conserved among dicots and monocots, which is consistent with the fact that NemaWater activates immune responses in tomato, sugar beet, tobacco, and rice. However, the corresponding PAMP molecule recognized by NILR1 has not been identified. The importance of PTI in immunity against PPNs has also been demonstrated in *Arabidopsis* PTI-deficient mutants (Teixeira et al., 2016; Mendy et al., 2017). The susceptibility of *Arabidopsis* to RKNs was enhanced in *bak1–5* and *bik1* mutants (Teixeira et al., 2016). BAK1 is a co-receptor for many PRRs inducing PTI, and in the BIK1 mutant, it is a required receptor-like cytoplasmic kinase for PTI signaling. *bak1–5* and *bak1–5 bkk1* (BKK1 is the closest homolog of BAK1) mutants are more susceptible to CNs (Mendy et al., 2017). Importantly, RKNs and CNs have multiple virulence effectors that are able to suppress PTI responses (Chen et al., 2013; Jaouannet et al., 2013; Lin et al., 2016; Chen et al., 2018; Naalden et al., 2018; Kud et al., 2019; Yang et al., 2019). PPN infections induce host-cell damage, thus they likely produce DAMP(s), which results in PTI induction. For example, CNs migrate intracellularly, thus their migration results in the release of oligogalacturonides (OGs) from plant cell walls. Fungal pathogens produce cell wall degrading enzymes like polygalacturonase (PG) to digest plant cell wall materials (D’Ovidio et al., 2004), and most plants have polygalacturonase inhibitor proteins (PGIPs) that attenuate pectin degradation by PGs, resulting in OG release. The released long-chain OGs activate PTI (Bishop et al., 1981; Hahn et al., 1981; Nothnagel et al., 1983; Benedetti et al., 2015). *Arabidopsis* has two PGIPs, PGIP1, and PGIP2, both of which are rapidly expressed during the migratory stage of CNs. A genetic study showed that PGIP1 activates plant camalexin and indole-glucosinolate pathways, thus attenuating CN infection (Shah et al., 2017). In addition, exogenous treatment with OGs enhances resistance against CNs. These results suggest that upon CN infection, *Arabidopsis* PGIP1 releases OGs, triggering PTI (Shah et al., 2017). Furthermore, CN infection induces ethylene production by the host, a signaling step that delays establishment of the syncytial-phase, indicating that damage-induced ethylene responses contribute to immunity against CNs (Marhavý et al., 2019). In contrast, there is as yet no clear evidence for damage-induced immunity against RKNs, which migrate intercellularly and are thus less-destructive than CNs. For example, neither PGIP1 nor PGIP2 are induced during the migratory stages of RKNs, and PGIP-mediated DAMP responses are not required for resistance against RKNs (Shah et al., 2017). Similarly, the loss of other DAMP receptors, PEPR1 and PEPR2 for plant elicitor peptides or DORN1 for extracellular ATP, fails to affect susceptibility to RKNs (Teixeira et al., 2016). However, it is possible that unknown DAMPs might be important for inducing immunity against RKNs, as PTI activation by exogenous application of known DAMPs is quite effective for suppressing the reproduction of RKNs (Lee et al., 2018).

NLR proteins also play critical roles in recognizing PPNs. NLRs involved in PPN recognition are mostly encoded by resistance (*R*) genes (Kaloshian et al., 2011). Well-studied *R* genes include tomato *Mi-1.2*, *Mi-9*, and *Hero-A*; potato *Gpa2* and *Gro1–4*; pepper *CaMi*; and prune *Ma* (Milligan et al., 1998; van der Vossen et al., 2000; Ernst et al., 2002; Paal et al., 2004; Chen et al., 2007; Jablonska et al., 2007; Claverie et al., 2011). *Mi-1.2*, *Mi-9*, *CaMi*, and *Ma* confer resistance against RKNs, while *Hero-A*, *Gpa2*, and *Gro1–4* provide resistance against CNs. *Gro1–4* and *Ma* encode TIR-NLRs, whereas the others encode CC-NLRs. Interestingly, Ma protein has a large and highly polymorphic C-terminal post-LRR region that is thought to be important for the recognition of PPNs (Claverie et al., 2011). Few examples of PPN avirulence factors recognized by NLRs are known. Gp-RBP-1, one of the secreted SP1a and RYanodine receptor (SPRY) domain (SPRYSEC) proteins from CN *Globodera pallida*, is an effector that induces hypersensitive response (HR)–cell death in the presence of GPA2 and Ran GTPase-activating protein 2 (RanGAP2) (Blanchard et al., 2005; Sacco et al., 2009). The proline residue at position 187 in the SPRY domain of Gp-RBP-1 is required for recognition by GPA2, whereas the virulent type Gp-RBP-1 variant allele has a mutation at this position, allowing it to avoid host recognition. Moreover, RanGAP2 interacts with the CC domain of GPA2 (Tameling and Baulcombe, 2007), suggesting that the RanGAP2-GPA2 complex is required for the recognition of the SPRY domain of Gp-RBP-1. Other example of an avirulence factor recognized by plants is Cg-1 in *M. javanica*, an RKN. The *Cg-1* gene is present in an *Mi*-*1.2*-avirulent population, but virulent RKN strains carry a deletion of *Cg-1* (Gleason et al., 2008; Gross and Williamson, 2011). Moreover, silencing of *Cg-1* in an avirulent strain increased virulence on *Mi-1.2*-containing tomato, suggesting a possible role for Cg-1 as a factor recognized by R protein Mi-1.2, although its signal transduction mechanism is unclear.

Surface-localized PRRs are also known to recognize PPN effectors. Venom allergen–like protein Gr-VAP1 from the CN *Globodera rostochiensis* interacts with apoplastic papain-like cysteine protease (PLCP) RCR3^pim^ in tomato to suppress host immunity (Lozano-Torres et al., 2012). However, Cf-2, a plasma membrane-localized *receptor-like protein* with extracellular LRRs, recognizes the interaction of Gr-VAP1 with RCR3^pim^, triggering HR-cell death in resistant hosts. Notably, *Cf-2* was originally identified as a resistance gene against the fungal pathogen *Cladosporium fulvum* (Rooney et al., 2005). Similar to Gr-VAP1, *C. fulvum* secretes AVR2, which interacts with and inhibits RCR3^pim^, and this interaction is recognized by Cf-2 protein. Thus, Cf-2 recognizes both fungal and nematode pathogens by monitoring RCR3^pim^.

## Secretion of Anti-Nematode Enzymes Into the Apoplast

**Figure 2:**
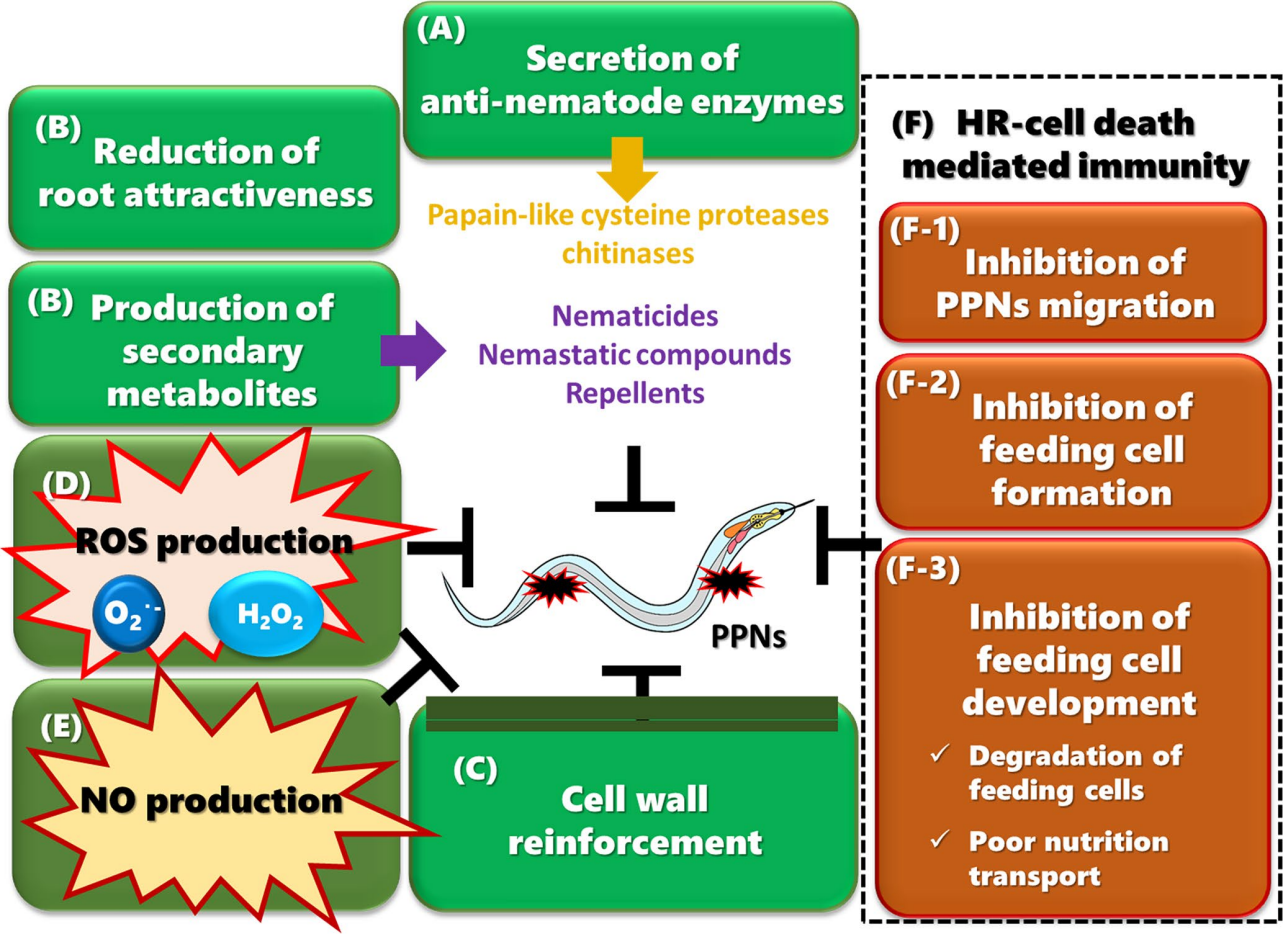
Multiple plant immune responses against PPNs **(A)** Plants secrete anti-nematode enzymes such as papain-like cysteine proteases (PLCPs) and chitinases into the apoplast to attack PPNs. **(B)** Resistant plants produce a wide range of secondary metabolites in response to PPN infection. Some metabolites inhibit egg hatching, suppress the motility of migrating PPNs, arrest growth and development, or kill nematodes. Plants may also reduce chemoattraction by secreting less amounts of attractants or more repellents. **(C)** Plants reinforce their cell walls by accumulating lignin, suberin, and callose, which strengthen the physical barrier to PPNs. **(D)** PPN infection induces the production of ROS, which may be directly toxic to PPNs. Hydrogen peroxide plays a role in cell wall cross-linking. ROS may also work as a transducing signal to activate immune responses and to control HR-cell death. **(E)** NO production is induced upon PPN infection and may play a role in JA-mediated defense responses, possibly through the production of protease inhibitor 2. **(F)** HR-cell death is crucial for limiting PPN movement and completing the life cycle. **(F-1)** HR-cell death occurs during penetration and migration of PPNs in cortical and epidermal tissues, contributing to inhibition of migration. **(F-2)** HR-cell death is induced in cells infected by RKNs or CNs, which inhibit the formation of feeding cells. **(F-3)** HR-cell death is also induced in cells surrounding feeding cells, often resulting in degeneration of feeding cells. Even if some feeding cells survive, the nutrient transport from surrounding tissues to the feeding cells is limited, causing a reduction in the number of eggs, and production of relatively more males. Some resistant plants induce the deterioration of feeding cells without any HR-cell death of surrounding cells.

The fact that the PPN effector Gr-VAP1 inhibits RCR3^pim^, a PLCP, implies that its enzymatic activity is important in immunity against PPNs (**Figure 2A**). Indeed, the absence of RCR3^pim^ homologs in *Arabidopsis* results in enhanced susceptibility to CN (Lozano-Torres et al., 2014). In addition to Gr-VAP1, Mc1194, an effector of RKN *Meloidogyne chitwoodi* targets another PLCP, RD21A in *Arabidopsis* (Davies et al., 2015b). Lack of RD21A leads to hyper-susceptibility to *M. chitwoodi*, showing that this PLCP also plays a positive role in immunity against RKN. However, it is not yet known how these PLCPs inhibit PPN infection.

Chitinases are also potentially important apoplastic enzymes in immunity against PPNs (**Figure 2A**). Upon fungal infection, plants often secrete chitinases, which degrade chitin in the fungal cell walls (Kumar et al., 2018; Pusztahelyi, 2018). In nematodes, chitin is the main component of the egg shell (Clarke et al., 1967; McClure and Bird, 1976; Perry and Trett, 1986) and makes up part of the pharyngeal lumen walls of *Caenorhabditis elegans* (Zhang et al., 2005), suggesting that chitinases may have anti-nematodal activity and thus contribute to immunity against PPNs. Consistent with this idea, chitinase activity and transcript levels are upregulated after PPN infection in resistant plants (Qiu et al., 1997; de-Deus Barbosa et al., 2009; Bagnaresi et al., 2013). However, there is currently no genetic evidence connecting plant chitinases to resistance against PPNs.

## Production of Anti-Nematode Compounds

Plants produce secondary metabolites in response to PPN invasion (**Figure 2B**). For instance, chlorogenic acid, a phenolic compound, is produced in various plants including solanaceous plants (Milne et al., 1965; Hung and Rohde, 1973; Pegard et al., 2005), carrots (Knypl et al., 1975), and rice (Plowright et al., 1996), suggesting a common defense response against PPN infection. Although the production of chlorogenic acid is well-correlated with PPN resistance levels, chlorogenic acid itself is only weakly nematicidal for *M. incognita* (Mahajan et al., 1985; D’Addabbo et al., 2013) with moderate activity against *Nacobbus aberrans*, a false root-knot nematode (López-Martínez et al., 2011). One possible explanation for this lack of correlation between response and effectiveness is that metabolized products of chlorogenic acid have higher nematicidal activity in the target organism, but those compounds may be unstable or highly toxic in plants. Chlorogenic acid can be hydrolyzed to quinic acid and caffeic acid, with the latter being further oxidized to orthoquinone, which is toxic to PPNs (Mahajan et al., 1985). However, the roles of caffeic acid and orthoquinone in resistance against PPNs need to be further established.

Another phenolic compound, phenylphenalenone anigorufone accumulates at the infection sites of the burrowing nematode *Radopholus similis* in a resistant banana cultivar (*Musa* sp.) (Dhakshinamoorthy et al., 2014; Hölscher et al., 2014). Anigorufone has high nematicidal activity because of the formation of large lipid–anigorufone complexes in the bodies of *R. similis*. Anigorufone is also known as an antifungal phytoalexin, and its synthesis is activated by infection with the pathogenic fungus *Fusarium oxysporum* (Luis et al., 1995). Interestingly, anigorufone also kills the human protozoan parasite *Leishmania* through the inhibition of succinate dehydrogenase in the mitochondrial respiratory complex II (Luque-Ortega et al., 2004). However, the toxic mechanism of anigorufone in PPNs and its relationship to the formation of large lipid–anigorufone complexes remains to be determined.

Flavonoids constitute a large class of secondary metabolites in plants. Some flavonoids play important roles in PPN resistance by functioning as nematicides, nemastatic compounds (which do not kill but inhibit their movement), repellents, or inhibitors of egg hatching (Chin et al., 2018). These flavonoids that have anti-nematodal activity mostly belong to the classes of flavonols (e.g., kaempferol, quercetin, myricetin), isoflavonoids, and pterocarpans (e.g., medicarpin, glyceollin). Kaempferol inhibits egg hatching of *R. similis* (Wuyts et al., 2006b). Kaempferol, quercetin, and myricetin are repellents and nemastatic to *M. incognita* juveniles (Wuyts et al., 2006b), and medicarpin also inhibits the motility of *Pratylenchus penetrans* in a concentration-dependent manner (Baldridge et al., 1998). Similarly, patuletin, patulitrin, quercetin, and rutin are nematicidal for infective juveniles of *Heterodera zeae*, a CN (Faizi et al., 2011). The synthesis of some flavonoids is also induced during infection in resistant plants. For instance, *M. incognita*–resistant soybean cultivars accumulate glyceollins, a group of soybean-specific prenylated pterocarpan phytoalexins that are expressed upon infection (Kaplan et al., 1980). Interestingly, glyceollin inhibits the motility of *M. incognita* (Kaplan et al., 1979; Kaplan et al., 1980). Glyceollin accumulation is also higher in CN-resistant soybean cultivars than in susceptible ones. One of the glyceollin isomers, glyceollin I accumulates in tissues adjacent to the head of the CN in resistant soybean roots (Huang and Barker, 1991), suggesting accumulation of glyceollin is spacio-temporally specific to the infection site.

Apart from phenolic compounds, other nematicidal chemicals are produced by several nematode-antagonistic plants, such as marigold and asparagus, which have been used for reducing nematode populations in soil. Marigold roots secrete α-terthienyl (Gommers and Bakker, 1988; Wang et al., 2007; Faizi et al., 2011), an oxidative stress-inducing chemical that effectively penetrates the nematode hypodermis and exerts nematicidal activity (Nivsarkar et al., 2001; Hamaguchi et al., 2019). Similarly, asparagus produces asparagusic acid, which inhibits hatching of two important CNs, *Heterodera glycines* and *G. rostochiensis* (Takasugi et al., 1975).

In Brassicaceae family plants, the broad spectrum antimicrobial isothiocyanates and indole glucosinolates are considered as anti-PPN compounds. Isothiocyanates effectively inhibit hatching of CNs and RKNs (Brown et al., 1997; Yu et al., 2005) and also have toxicity to RKNs and the semi-endoparasitic nematode *Tylenchulus semipenetrans* (Zasada and Ferris, 2003). In *Arabidopsis*, the synthesis of camalexin, an indole alkaloid glucosinolate-type phytoalexin, is catalyzed by three cytochrome P450-dependent monooxygenases, CYP79B2, CYP79B3 (Hull et al., 2000; Mikkelsen et al., 2000; Bak et al., 2001; Mikkelsen et al., 2004), and PAD3 (phytoalexin-deficient 3, CYP71B15). Double mutants *cyp79b2/b3* which do not accumulate indolic glucosinolates are more susceptible to CNs (Shah et al., 2017), while *pad3*, camalexin-deficient mutants are more susceptible to RKNs than wild type (Teixeira et al., 2016). These results suggest that some indole glucosinolates including camalexin have some inhibitory effects on PPNs, but there have so far been no reports of direct toxicity of indolic glucosinolates on PPNs.

In addition to nematicides and nemastatic compounds, interruption of PPN chemotaxis may also be an effective plant response for inhibiting or limiting PPN infection. Ethylene, which is normally produced after wounding as well as during pathogen invasion, reduces PPN attraction to the root (Booker and DeLong, 2015; Guan et al., 2015; Marhavý et al., 2019). An ethylene-overproducing *Arabidopsis* mutant is less attractive for PPNs, and attractiveness is greater in plants treated with ethylene-synthesis inhibitors or in ethylene-insensitive mutants (Fudali et al., 2013; Hu et al., 2017). These results suggest that PPN infection induces ethylene production, which possibly prevents secondary PPN invasion by reducing attractiveness. The reduced attractiveness could be due to a reduction in attractant secretion or an increase in repellents. However, the molecular basis of the attractiveness for PPNs is still largely unknown. Several groups have tried to identify RKN attractants from root tips (Čepulytė et al., 2018) and seed-coat mucilage (Tsai et al., 2019). The identification of chemoattractants and chemorepellents may offer some insight into how plants respond to nematodes in the rhizosphere both before and during PPN infection.

## Reinforcement of Cell Wall as a Physical Barrier

Since all PPNs must penetrate the cell wall for feeding, reinforcement of cell wall structure has been implicated as an effective defense as a physical barrier (**Figure 2C**). For instance, PPN infection often induces accumulation of lignin in resistant plants (Balhadère and Evans, 1995a; Balhadère and Evans, 1995b; Andres et al., 2001; Dhakshinamoorthy et al., 2014). Moreover, *Arabidopsis* mutants with increased levels of syringyl lignin have reduced *M. incognita* reproduction rates (Wuyts et al., 2006a). These results suggest that lignin accumulation in roots is an effective antagonist to PPN infection.

The effectiveness of lignin accumulation for suppressing nematode infection is also supported by plant immune inducers such as β-aminobutyric acid (BABA), thiamine, and sclareol. BABA, a non-protein amino acid, has broad efficacy against viruses, bacteria, fungi, and oomycetes in various plants (Alexandersson et al., 2016; Cohen et al., 2016). Treatment with BABA inhibits RKN invasion, delays giant cell formation, and retards RKN development. Interestingly, BABA induces lignin accumulation in roots, and callose accumulation in galls (Ji et al., 2015). Thiamine (vitamin B1) treatment also induces lignin accumulation in roots; enhances the expression of phenylalanine ammonia-lyase, a key enzyme of the phenylpropanoid biosynthesis pathway; reduces PPN penetration; and delays PPN development (Huang et al., 2016). An inhibitor of phenylalanine ammonia-lyase suppresses thiamin-mediated immunity, indicating that activation of the phenylpropanoid pathway with subsequent lignin accumulation is important for thiamin-mediated immunity against nematodes. Treatment with sclareol, an antimicrobial compound with activity against some plant-pathogenic bacteria and fungi (Bailey et al., 1975; Kennedy et al., 1992; Seo et al., 2012), also induces lignin accumulation and suppresses RKN penetration (Fujimoto et al., 2015). Importantly, an *Arabidopsis* mutant of *cinnamoyl-coA reductase* (*ccr2*) defective in lignin accumulation cannot induce sclareol-mediated suppression of RKN penetration, suggesting that lignin accumulation is important for the sclareol-mediated immunity.

Similar to lignin accumulation, callose deposition and suberin accumulation may also reinforce cell walls and contribute to immunity against PPNs. The RKN *Meloidogyne naasi* induces callose deposition at an early infection stage, and suberin accumulation at a later stage in the resistant grass plant *Aegilops variabilis* (Balhadère and Evans, 1995a; Balhadère and Evans, 1995b). Infection of *Arabidopsis* by RKN or CN also induces transcriptional activation of suberin biosynthesis genes at the site of infection (Holbein et al., 2019). Overexpression of the transcription factor RAP2.6 in *Arabidopsis* leads to enhanced callose deposition at syncytia and results in higher resistance to CN (Ali et al., 2013). RAP2.6 is strongly downregulated in syncytia compared to uninfected root; therefore, it is possible that CN suppresses RAP2.6 expression to inhibit callose deposition within syncytia.

Lignin and suberin in suberin lamellae and casparian strips at the endodermis are also important basal physical barriers to RKNs. RKNs are not able to directly cross the endodermis because of the reinforcement of cell walls by suberin lamellae and casparian strips (Wyss et al., 1992; Abad et al., 2009). Indeed, *Arabidopsis* mutants defective in casparian strips are more susceptible to RKNs (Holbein et al., 2019).

## Reactive Oxygen Species (ROS)

The rapid production of ROS, such as superoxide anion and hydrogen peroxide, is a conserved signaling response across kingdoms, and in plants, it is induced at an early stage of PPN infection (**Figure 2D**). ROS have direct antimicrobial properties but also serve as signaling molecules to activate additional and complementary immune outputs such as strengthening cell walls by cross-linking polymers, amplifying and propagating intra- and intercellular defense signals, and regulating HR-cell death (Torres et al., 2006; Kadota et al., 2015). Resistant tomato plants carrying the *Mi-1.2* gene respond to RKN infection with a strong and prolonged induction of ROS. On the other hand, susceptible tomato plants have weak and transient ROS induction in response to nematode infection (Melillo et al., 2006; Melillo et al., 2011; Zhou et al., 2018). Similarly, strong ROS production is induced in *Arabidopsis* roots during incompatible interactions with the soybean CN *H. glycines* (Waetzig et al., 1999). Histochemical studies showed that hydrogen peroxide accumulates in the apoplast after infection of the avirulent RKNs or CNs (Waetzig et al., 1999; Melillo et al., 2006).

The plasma membrane-bound NADPH oxidase respiratory burst oxidase homologs (RBOHs) are important for the production of apoplastic ROS (Kadota et al., 2015). In tomato, whitefly-induced 1 (WFI1), an RBOH homolog, is required for Mi-1.2-mediated ROS accumulation during RKN infection. Consistently, HSFA1, a class-A heat-shock factor that regulates *Wfi1* transcription by binding to the *Wfi1* promoter, is also critical for *Mi-1.2*-mediated ROS production (Zhou et al., 2018). In *Arabidopsis*, which has 10 RBOHs, RBOHD is the primary source of ROS production during PTI and NLR-triggered immunity. RBOHF may also work redundantly with RBOHD in some responses, because the *rbohD rbohF* double mutant has a stronger defense response phenotype against bacterial pathogens (Torres and Dangl, 2005; Torres et al., 2006). Similarly, *rbohD rbohF* produces more galls after RKN infection than the wild type (Teixeira et al., 2016), indicating a positive role for RBOHD and RBOHF ROS production in immunity against RKNs. Interestingly, fewer CNs develop in *rbohD rbohF* double mutant, suggesting that CNs require a different level of ROS control by RBOH for successful establishment of infection. Furthermore, *rbohD rbohF* exhibits larger regions of HR-cell death and less syncytium formation upon CN infection, suggesting that CNs utilize RBOHD- and RBOHF-mediated ROS to suppress HR-cell death in the host (Siddique et al., 2014).

To protect themselves from the toxicity of produced ROS by the host, endoparasitic nematodes may have evolved a number of antioxidant enzymes on their surface and in the hypodermis (Henkle-Dührsen and Kampkötter, 2001). For example, both CNs and RKNs produce peroxiredoxins; some of the most abundant detoxifying antioxidant enzymes, which remove hydrogen peroxides from the apoplast of host plants by thioredoxin (TRX) cysteine thiol-disulfide exchange (Robertson et al., 2000; Henkle-Dührsen and Kampkötter, 2001; Dubreuil et al., 2011). PRX2.1, a clade B peroxiredoxin in *M. incognita*, is expressed upon infection, and knock-down of the gene reduces resistance against oxidative stress, resulting in fewer galls. This interaction suggests a critical role for PRX2.1 in infection. CNs also secrete GPX-1, a glutathione peroxidase variant, from the hypodermis to scavenge host-derived ROS, thereby protecting external cell membranes from oxidation (Jones et al., 2004). *M. incognita* glutathione-S-transferases are delivered into the host apoplast to detoxify the products of oxidative stress (Dubreuil et al., 2007). Indeed, freshly hatched infective juveniles of *M. incognita* are much more resistant to exogenous treatment with hydrogen peroxide than *C. elegans* (Isermann et al., 2004).

Another PPN strategy for protection against host ROS is to activate the host ROS-scavenging system by the secretion of virulence effectors. For example, CN effector 10A06 interacts with host spermidine synthase 2 and increases spermidine content in infected tissues (Hewezi et al., 2010). Spermidine in higher concentrations functions as a ROS scavenger, and in lower concentrations, it indirectly decreases oxidative stress by activating cellular antioxidant systems (Kasukabe et al., 2004). Indeed, ectopic expression of 10A06 in *Arabidopsis* increases the expression of several genes encoding antioxidant enzymes. Similarly, MjTTL5, a virulence effector from *M. javanica* interacts with the *Arabidopsis* ferredoxin:TRX reductase (FTR) catalytic subunit (FTRc) in plastids (Lin et al., 2016). FTR activates TRXs in chloroplasts or plastids by receiving reducing equivalents from reduced ferredoxin (Balmer et al., 2006; Kirchsteiger et al., 2012). The interaction of MjTTL5 with FTRc drastically increases host ROS-scavenging activity, thus modulating the plant immune reaction. Because peroxiredoxins use TRX to reduce hydrogen peroxide (Broin et al., 2002; Kotze, 2003), it is possible that FTRc works in part with peroxiredoxins by providing reduced TRX to lower ROS production in plants.

## Nitric Oxide (NO) and Protease Inhibitor-Based Immunity

NO is an essential signaling molecule that has multiple functions in plants (Delledonne et al., 1998; Torres et al., 2006; Bellin et al., 2013; Mur et al., 2013; Scheler et al., 2013) (**Figure 2E**). After infection with *M. incognita*, resistant tomato plants carrying *Mi-1.2* produce more NO than susceptible cultivars (Melillo et al., 2011). Application of an exogenous NO donor, sodium nitroprusside (SNP), to susceptible tomato plants significantly enhances immunity against RKNs (Zhou et al., 2015). Treatment with SNP reduces the number of egg masses and restores the growth inhibition associated with PPNs, suggesting that NO plays a positive role in immunity. NO may be involved in the JA-dependent RKN defense pathway, as an NO scavenger partially inhibitis JA-induced RKN defense responses. Moreover, the inhibition of JA biosynthesis by chemical inhibitors significantly increased susceptibility to RKNs, but resistance was effectively restored by exogenous SNP application. Because both JA- and SNP-induced RKN defense responses are compromised by silencing *protease inhibitor 2* (*PI2*), the NO- and JA-pathways likely converge to induce immunity against PPNs (Zhou et al., 2015). However, it remains unclear which proteases PI2 inhibits. Since PPNs use a variety of proteases for their virulence and for their development (Urwin et al., 1997; Neveu et al., 2003), these activities can be inhibited by PI2. Interestingly, heterologous expression of various protease inhibitors, including trypsin inhibitors and cysteine protease inhibitors, confer resistance against PPNs, showing the effectiveness of protease inhibitor-based immunity against PPNs (Hepher and Atkinson, 1992; Urwin et al., 2000; Urwin et al., 2003).

## HR-Cell Death-Based Inhibition of Nematode Development

HR-cell death, a type of programmed cell death that is induced after the invasion of avirulent pathogens to prevent the spread of biotrophic pathogens (Huysmans et al., 2017), also plays a crucial role in PPN immunity (**Figure 2F**). HR-cell death has been observed at three different phases of PPN infection in resistant plants: (1) in the cortex and epidermis during PPN penetration and migration (Hung and Rohde, 1973; Thomason et al., 1976; Finetti Sialer, 1990; Balhadère and Evans, 1995b; Pegard et al., 2005; Proite et al., 2008; Albuquerque et al., 2010; Khallouk et al., 2011; Cabasan et al., 2014; Davies et al., 2015a), (2) in vascular tissues during the initiation of feeding cell formation (Paulson and Webster, 1972; Melillo et al., 2006), and (3) in cells adjacent to developing feeding cells (Kim et al., 1987; Rice et al., 1987; Sobczak et al., 2005; Kim et al., 2010; Kim et al., 2012; Cabasan et al., 2014; Seo et al., 2014; Ye et al., 2017).

During PPN penetration and migration, cell death is also often observed in susceptible plants, but it is less rapid and less frequent than in resistant varieties (Endo and Veech, 1970; Thomason et al., 1976; Sobczak et al., 2005). HR-cell death may inhibit nematode migration, but it is not clear if HR-cell death stops PPN movement directly, *or indirectly by releasing* nemastatic or nematicidal chemicals or DAMPs to activate other immune responses. HR-cell death is also induced during the initiation of feeding cell development. For instance, *Mi-1.2*-resistant tomato plants induce HR-cell death during the RKN induction of giant cells, thus inhibiting the development of feeding cells (Paulson and Webster, 1972; Melillo et al., 2006). Another possible function of HR-cell death is to create a physical gap between feeding cells and surrounding cells to block nutrient and water supplies. For example, in resistant tomato lines carrying the *Hero* gene, potato CN (*G. rostochiensis*) makes syncytia, but HR-cell death is induced in surrounding cells, which resulted in the separation of the syncytium from stelar conductive tissues (Sobczak et al., 2005). Disconnection of feeding cells from surrounding tissue also occurs in resistant plants after infection with RKNs (Seo et al., 2014; Ye et al., 2017). Disassociation of surrounding tissue leads to poor nutrient supply, thereby inhibiting growth or causing the death of feeding cells, reducing fecundity in females, and increasing male development (Acedo et al., 1984; Rice et al., 1987; Kouassi et al., 2004; Sobczak et al., 2005). Increased male development coincides with a reduced number of females, resulting in the reduction of PPN eggs. In some resistant plants, death of feeding cells is also induced without HR-cell death of surrounding cells. For example, death of syncytia is induced in resistant soybeans (Yan and Baidoo, 2018), and deterioration of giant cells is induced in resistant cowpea carrying *Rk* gene without typical HR-cell death in surrounding cells (Das et al., 2008). These differences in HR-cell death initiation site may depend on the specific expression pattern of host *R* genes (Yan and Baidoo, 2018) and PPN effectors.

The importance of HR-cell death is supported by the observation that both RKNs and CNs have effectors that suppress HR-cell death. The *M. incognita* effector MiISE5, a zinc-finger protein, suppresses HR-cell death induced by the non-host bacterial pathogen, *Burkholderia glumae* in *N. benthamiana*, possibly through reprogramming of the host transcriptome (Shi et al., 2018). The RKN effector MeTCTP from *Meloidogyne enterolobii* also suppresses HR-cell death triggered by the mouse pro-apoptotic protein, Bcl2 associated X protein (Zhuo et al., 2017). CNs also have HR-cell death suppression effectors such as SPRYSEC effectors (Ali et al., 2015b), RHA1B, an E3 ubiquitin ligase (Kud et al., 2019), and GrEXPB2, an expansin-like protein (Ali et al., 2015a). However, these CN effectors do not specifically inhibit HR-cell death but also inhibit other defense responses.

## Conclusions and Future Directions

**Figure 3:**
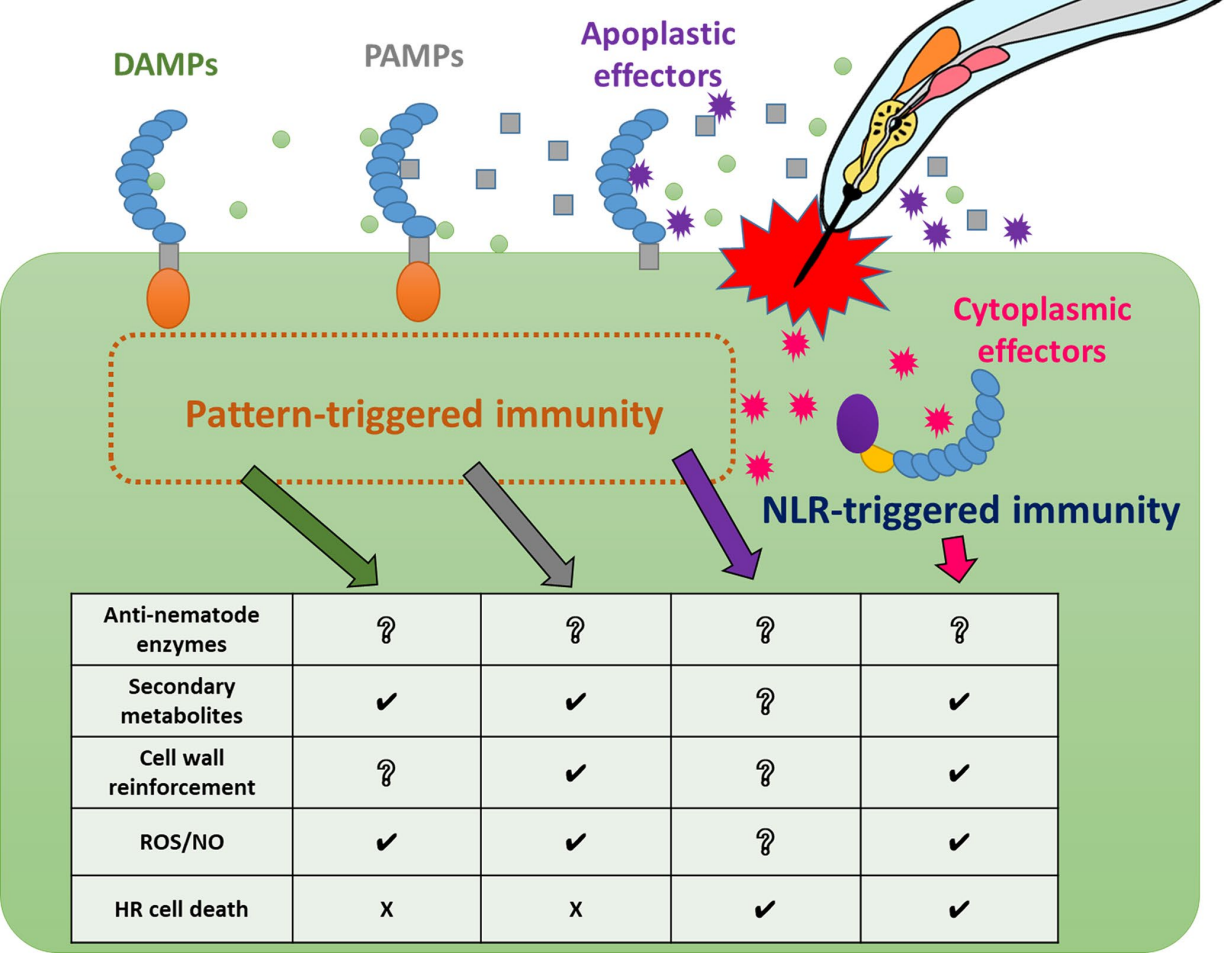
Relationships between nematode recognition and immune responses Plants activate pattern-triggered immunity and NLR-triggered immunity against PPN infection using different immune receptors. These receptors trigger a variety of defense responses. Some immune responses, such as ROS/NO production, are induced in common by some immune receptors with different kinetics, while other responses, such as cell death, are induced by specific immune receptors.

As a result of the identification of several NLR-type and PRR-type receptors involved in immunity against PPNs, we have gradually begun to understand how plants recognize and respond to nematode infection at the molecular level. However, PPN effectors and PAMPs are still largely unknown, and the corresponding receptors remain unidentified. Similarly, various immune responses against nematodes in a wide range of resistant crop and model plants have been recognized (**Figure 2** and **Supplementary Table 1**), but there is still much that is unknown between the phenomena of PPN recognition and the triggering of specific immune responses (**Figure 3**). Thus, significant challenges for future research in the field of plant and nematode interactions would be to identify immune receptor-ligands pairs (PAMPs, DAMPs, and effectors), to clarify the molecular bases of signaling pathways leading to individual immune responses, to understand the interactions of these components and signaling pathways in PPN immunity, and to identify the molecular components that define host specificity. Loss of significant agricultural productivity in a burgeoning global population goes beyond monetary losses. The absence of truly effective strategies for controlling nematode populations and infection has serious and worsening consequences for sustainable agriculture. Understanding the molecular mechanisms of PPN recognition and immune signaling networks will provide a knowledge base for much-needed PPN disease control strategies in the future.

## Author Contributions

All authors listed have made a substantial, direct and intellectual contribution to the work, and approved it for publication.

## Funding

This work was supported by Grant-in-Aid for JSPS Fellows Grant number JP19J00655 (to KSa), and MEXT/JSPS KAKENHI Grant Number, JP16H06186, JP16KT0037 and JP19H02962 (to YK), JP15H05959 and JP17H06172 (to KSh).

## Conflict of Interest

The authors declare that the research was conducted in the absence of any commercial or financial relationships that could be construed as a potential conflict of interest.

## Abbreviations

BABA, β-aminobutyric acid; CC, coiled-coil; CN, Cyst nematode; FTR, ferredoxin:thioredoxin reductase; HR, hypersensitive response; JA, jasmonic acid; LRR, leucine-rich repeat; NLR, nucleotide-binding domain leucine-rich repeat; NO, nitric oxide; OG, oligogalacturonides; PAMP, pathogen-associated molecular pattern; PG, polygalacturonase; PGIP, polygalacturonase inhibitor proteins; PLCP, papain-like cysteine protease; PPN, plant parasitic nematode; PRR, pattern recognition receptor; PTI, pattern-triggered immunity; RBOH, respiratory burst oxidase homolog; RKN, root-knot nematode; RLK, receptor-like kinase; ROS, reactive oxygen species; SNP, sodium nitroprusside; SPRYSEC, secreted SP1a and ryanodine receptor (SPRY) domain; TIR, toll-interleukin 1 receptor; TRX, thioredoxin.
