# Supplementary material for: Plant Immune Responses to Parasitic Nematodes
Source: Front Plant Sci. 2019 Sep 26;10:1165. doi: 10.3389/fpls.2019.01165 (PMC6775239; doi:10.3389/fpls.2019.01165)
Supplement: Supplementary file 1 [file Table_1.docx]

**Supplementary Table 1. Examples of plant responses to PPN infection**

| Resistant plant | Gene (cultivar)* | PPNs | Responses in plants | References |
| --- | --- | --- | --- | --- |
| Solanaceae | | | | |
| Tomato  *(Solanum lycopersicum)* | *Mi-1.2* | *Meloidogyne incognita* | **ROS production, NO production, HR-cell death (inhibition of migration and feeding cell formation),** | Paulson and Webster, 1972; Melillo et al., 2006, 2011; Zhou et al., 2018 |
|  | *Hero* | *Globodera rostochiensis* | **HR-cell death (inhibition of feeding cell development)** | Sobczak et al., 2005 |
|  | (Nemard)  (Hawaii 7153) | *Pratylenchus penetrans*  *M. incognita* | **Production of secondary metabolites (cholorogenic acid and its oxidized products), HR-cell death (inhibition of migration)** | Hung and Rohde., 1973 |
| Potato  (*Solanum tuberosum*) | *H1* | *G. rostochiensis* | **HR-cell death (inhibition of feeding site development)** | Rice et al., 1987 |
|  | *R_Mc1(blb)_* | *M. chitwoodi* | **ROS production, HR-cell death (inhibition of migration)** | Davies et al., 2015a |
|  | (B-25) | *Nacobbus aberrans* | **HR-cell death (inhibition of migration)** | Finetti Sialer, 1990 |
| Pepper  (*Capsicum annuum*) | *Me*_1_ | *M. incognita*  *M. arenaria*  *M. javanica* | **HR-cell death (inhibition of migration) (*M.a*, *M.j*), HR-cell death (inhibition of feeding site development) (*M.i*)** | Hendy et al., 1985;Bleve-Zacheo et al., 1998 |
|  | *Me*_3_ | *M. incognita*  *M. arenaria*  *M. javanica* | **HR-cell death (inhibition of migration) (*M.a*, *M.j*, *M.i*), HR-cell death (inhibition of feeding site development) (*M.a*)** | Hendy et al., 1985;Bleve-Zacheo et al., 1998 |
|  | *Me*_7_ | *M. incognita*  *M. arenaria*  *M. javanica* | **Production of secondary metabolites (chlorogenic acid and unidentified phenolics), HR-cell death (inhibition of migration)** | Pegard et al., 2005 |
| *Nicotiana repanda* |  | *M. incognita*  *M. arenaria*  *M. javanica*  *M. hapla* | **Production of secondary metabolites (chlorogenic acid), HR-cell death (inhibition of migration)** | Milne et al., 1965 |
| *Solanum torvum* |  | *M. incognita* | **Production of secondary metabolites (Sesquiterpenoids)**  **Enzyme-based immunity (chitinase)** | Bagnaresi et al., 2013 |
| Cucurbitaceae | | | | |
| Horned melon  (*Cucumis metuliferus*) | (PI482443) | *M. incognita* | **HR-cell death (inhibition of feeding cell development)** | Ye et al., 2017 |
| *C. metuliferus*  *C. ficifolius* | (C-701)  (C-779) | *M. incognita* | **Inhibition of feeding cell development (without HR-cell death in surrounding tissue)** | Fassuliotis, 1970 |
| Fabaceae | | | | |
| Cowpea  (*Vigna unguiculate*) | *Rk* | *M. incognita* | **Inhibition of feeding cell development (without HR-cell death in surrounding tissue)** | Das et al., 2008 |
| Soybean  (*Glycine max*) | *Rhg1 and Rhg4* | *Heterodera glycines* | **Inhibition of feeding cell development (without HR-cell death in surrounding tissue)** | Kim et al., 1987, 2010, 2012 |
|  | *Rhg1* | *H. glycines* | **HR-cell death (inhibition of feeding cell development)** | Kim et al., 1987, 2010, 2012 |
|  | (Bryan) | *M. incognita* | **Enzyme-based immunity (chitinase)** | Qiu et al., 1997 |
|  | (Centennial) | *M. incognita* | **Production of secondary metabolites (glyceollin), HR-cell death (inhibition of migration)** | Kaplan et al., 1979, 1980 |
|  |  | *H. glycines* | **Production of secondary metabolites (glyceollin)** | Huang and Barker, 1991 |
|  | (PI209332, PI89772) | *H. glycines* | **HR-cell death (inhibition of feeding cell development)** | Acedo et al., 1984 |
|  | (Pickett) | *H. glycines* | **HR-cell death (inhibition of feeding cell development)** | Endo and Veech, 1970 |
| Lima bean  (*Phaseolus lunatus*) | (Fordhook 242) | *P. scribneri* | **Production of secondary metabolites (coumesterol), HR-cell death (inhibition of migration)** | Thomason et al., 1976; Rich et al., 1977 |
| Wild peanut  (*Arachis stenosperma*) | (V10309) | *M. arenaria* | **HR-cell death (inhibition of migration)** | Proite et al., 2008 |
| Malvaceae | | | | |
| Cotton  (*Gossypium hirsutum*) | (Auburn 623 RNR) | *M. incognita* | **Production of secondary metabolites (terpenoid aldehydes)** | Veech and McClure, 1977;Veech, 1979 |
| *Gossypium hirsutum* | (TX-25) | *M. incognita* | **Production of secondary metabolites (phenolics, based on microscopic observation), HR-cell death (inhibition of feeding cell development)** | Mota et al., 2013 |
| *Gossypium barbadense* | (CIR1348) | *M. incognita* | **Production of secondary metabolites (phenolics, based on microscopic observation), HR-cell death (inhibition of migration and feeding cell development)** | Mota et al., 2013 |
| Amaranthaceae | | | | |
| Sugar beet  (*Beta vulgaris*) | *Hs1^pro-1^* | *H. schachtii* | **Inhibition of feeding cell development (without HR-cell death in surrounding tissue)** | Holtmann et al., 2000 |
|  | (51501)  Hybrid of *B. vulgaris* x *B. procumbens* | *H. schachtii* | **HR-cell death (Inhibition of feeding cell development)** | Yu and Steele, 1981 |
| Musaceae | | | | |
| Banana  (*Musa spp.*) | (Yanginobi km5, Long Tavoy, Saba, Pisang jari buaya, and Calcutta 4) | *Radopholus similis* | **Production of secondary metabolites (phenolics), Cell wall reinforcement (lignin)** | Valette et al., 1998;Wuyts et al., 2007; Dhakshinamoorthy et al., 2014 |
|  | (Yangambi km5) | *R. similis* | **Production of secondary metabolites (anigorufone)** | Hölscher et al. 2014 |
| Brassicaceae | | | | |
| Radish  (*Raphanus sativus*) | (Pegletta) | *H. schachtii* | **Inhibition of feeding cell development (without HR-cell death in surrounding tissue)** | Grymaszewska and Golinowski, 1998 |
| *Arabidopsis thaliana* |  | *M. chitwoodi* | **Enzyme-based immunity (PLCP)** | Davies et al., 2015b |
|  |  | *M. incognita* | **Production of secondary metabolites (camalexin)** | Teixeira et al., 2016 |
|  |  | *M. incognita* | **Cell wall reinforcement (lignin)** | Wuyts et al., 2006a |
|  |  | *H. schachtii* | **Cell wall reinforcement (callose)** | Ali et al., 2013 |
|  |  | *H. glycines* | **ROS production** | Waetzig et al., 1999 |
| Poaceae (syn. Gramineae) | | | | |
| African rice  (*Oryza glaberrima*) | (CG14) | *M. graminicola* | **Production of secondary metabolites (phenolics, based on microscopic observation), HR-cell death (inhibition of migration and feeding cell development)** | Cabasan et al., 2014 |
| Rice  (Oryza spp.) | Rayada 16-06(I) | *Ditylenchus angustus* | **Production of secondary metabolites (chlorogenic acid)** | Plowright et al., 1996 |
| Wild grasses  (*Aegilops variabilis*) | (x^8^ and n°1) | *M. naasi* | **Production of secondary metabolites (oxidized phenols, based on microscopic observation), Cell wall reinforcement (callose, suberin), HR-cell death (inhibition of migration)** | Balhadère and Evans, 1995a, b |
| Wild barley  (*Hordeum chilense*) | (PI283375) | *M. naasi* | **Production of secondary metabolites (oxidized phenols, based on microscopic observation), Cell wall reinforcement (lignin, suberin), HR-cell death (inhibition of migration)** | Balhadère and Evans, 1995a, b |
| Triticum aestivum/Aegilops ventricosa introgression line (H-93-8) | *Cre2* | *H. avenae* | **Cell wall reinforcement (lignin), HR-cell death (inhibition of feeding cell development)** | Andres et al. 2001 |
| Rubiaceae | | | | |
| *Coffea arabica*  (Coffee plants) | *Mex-1* | *M. exigua* | **Production of secondary metabolites (phenolics, based on microscopic observation), HR-cell death (inhibition of migration and feeding cell development)** | Anthony et al., 2005 |
| *Coffea arabica* | (UFV408-28) | *M. incognita* | **Production of secondary metabolites (phenolics, based on microscopic observation), HR-cell death (inhibition of migration)** | Albuquerque et al., 2010 |
| *Coffea canephora* | (clone 14) | *M. incognita* | **Production of secondary metabolites (phenolics, based on microscopic observation), HR-cell death (inhibition of migration and feeding site development)** | Lima et al., 2015 |
| Apiaceae | | | | |
| Carrot  (*Daucus carota* var. *sativus*) | (L 1213(1)) | *M. incognita* | **HR-cell death (inhibition of feeding cell development)** | Seo et al., 2014 |
| Myrtaceae | | | | |
| Yellow guava  *Psidium cattleianum*  Costa Rican guava  *Psidum friedrichsthalianum* |  | *M. enterolobii* | **HR-cell death (inhibition of feeding cell development)** | Freitas et al., 2014 |
| Rosaceae | | | | |
| *Prunus* spp. | *Ma* | *M. incognita* | **Production of secondary metabolites (phenolics, based on microscopic observation), HR-cell death (inhibition of migration)** | Khallouk et al., 2011 |

*If a resistance gene is not provided in references, information of cultivars is shown in parentheses.

**References (cited only in supplementary table 1)**

Anthony, F., Topart, P., Martinez, A., Silva, M., and Nicole, M. (2005). Hypersensitive-like reaction conferred by the *Mex-1* resistance gene against *Meloidogyne exigua* in coffee. *Plant Pathol* 54**,** 476-48210.1111/j.1365-3059.2005.01239.x.

Bleve-Zacheo, T., Bongiovanni, M., Melillo, M., and Castagnone-Sereno, P. (1998). The pepper resistance genes *Me1* and *Me3* induce differential penetration rates and temporal sequences of root cell ultrastructural changes upon nematode infection. *Plant Science* 133**,** 79-9010.1016/S0168-9452(98)00021-1.

Fassuliotis, G. (1970). Resistance of *Cucumis* spp. to the root-knot nematode, *Meloidogyne incognita acrita*. *J Nematol* 2**,** 174-178

Freitas, V., Correa, V., Motta, F., Sousa, M., Gomes, A., Carneiro, M., Silva, D., Mattos, J., Nicole, M., and Carneiro, R. (2014). Resistant accessions of wild *Psidium* spp. to *Meloidogyne enterolobii* and histological characterization of resistance. *Plant Pathol* 63**,** 738-74610.1111/ppa.12149.

Grymaszewska, G., and Golinowski, W. (1998). Structure of syncytia induced by *Heterodera schachtii* Schmidt in roots of susceptible and resistant radish (*Raphanus sativus* L. var. *oleiformis*). *Acta Societatis Botanicorum Poloniae* 67**,** 207-216

Hendy, H., Dalmasso, A., and Cardin, M. (1985). Differences in resistant *Capsicum annuum* attacked by different *Meloidogyne* species. *Nematologica* 31**,** 72-7810.1163/187529285X00094.

Holtmann, B., Kleine, M., and Grundler, F. (2000). Ultrastructure and anatomy of nematode-induced syncytia in roots of susceptible and resistant sugar beet. *Protoplasma* 211**,** 39-5010.1007/BF01279898.

Lima, E.A., Furlanetto, C., Nicole, M., Gomes, A.C., Almeida, M.R., Jorge-Júnior, A., Correa, V.R., Salgado, S.M., Ferrão, M.A., and Carneiro, R.M. (2015). The multi-resistant reaction of drought-tolerant coffee 'Conilon clone 14' to *Meloidogyne* spp. and late hypersensitive-like response in *Coffea canephora*. *Phytopathology* 105**,** 805-81410.1094/PHYTO-08-14-0232-R.

Mota, F.C., Alves, G.C.S., Giband, M., Gomes, A.C.M.M., Sousa, F.R., Mattos, V.S., Barbosa, V.H.S., Barroso, P.a.V., Nicole, M., Peixoto, J.R., Rocha, M.R., and Carneiro, R.M.D.G. (2013). New sources of resistance to *Meloidogyne incognita* race 3 in wild cotton accessions and histological characterization of the defence mechanisms. *Plant Pathol* 62**,** 1173-118310.1111/ppa.12022.

Rich, J.R., Keen, N.T., and Thomason, I.J. (1977). Association of coumestans with the hypersensitivity of Lima bean roots to *Pratylenchus scribneri*. *Physiol Plant Pathol* 10**,** 105-11610.1016/0048-4059(77)90014-5.

Valette, C., Andary, C., Geiger, J.P., Sarah, J.L., and Nicole, M. (1998). Histochemical and Cytochemical Investigations of Phenols in Roots of Banana Infected by the Burrowing Nematode Radopholus similis. *Phytopathology* 88**,** 1141-114810.1094/PHYTO.1998.88.11.1141.

Veech, J.A. (1979). Histochemical localization and nematoxicity of terpenoid aldehydes in cotton. *J Nematol* 11**,** 240-246

Veech, J.A., and Mcclure, M.A. (1977). Terpenoid aldehydes in cotton roots susceptible and resistant to the root-knot nematode, Meloidogyne incognita. *J Nematol* 9**,** 225-229

Wuyts, N., Lognay, G., Verscheure, M., Marlier, M., De Waele, D., and Swennen, R. (2007). Potential physical and chemical barriers to infection by the burrowing nematode Radopholus similis in roots of susceptible and resistant banana (Musa spp.). *Plant Pathology* 56**,** 878-89010.1111/j.1365-3059.2007.01607.x.

Yu, M.H., and Steele, A.E. (1981). Host-parasite interaction of resistant sugarbeet and *Heterodera schachtii*. *J Nematol* 13**,** 206-212
